# Suppression of Frequent Ventricular Ectopy in a Patient with Hypertrophic Heart Disease with Ranolazine: A Case Report

**Published:** 2011-05-01

**Authors:** David K Murdock, Jeffrey W Kaliebe

**Affiliations:** 1 CaRE Foundation, Inc; 2 Aspirus Cardiovascular Associates

**Keywords:** Ventricular ectopy, PVCs, ranolazine, triggered activity, cardiac arrhythmias

## Abstract

**Background:**

Pro-arrhythmic concerns with most anti-arrhythmic agents in patients with significant left ventricular hypertrophy (LVH) limits options when anti-arrhythmic therapy is indicated. Ranolazine, an anti-anginal agent which inhibits late Na+ currents, indirectly causes a decrease in diastolic cardiomyocyte Ca++ levels producing an energy sparing effect. Ranolazine also inhibits triggered activity in animal studies and has anti-arrhythmic properties in patients with ischemic heart disease. Here we report the dramatic anti-arrhythmic effects of ranolazine in a patient with frequent ventricular and supraventricular ectopy in the setting of hypertrophic heart disease without significant coronary artery disease.

**Methods:**

A 72 year old hypertensive patient with palpitations and significant exercise intolerance due to dyspnea was evaluated with echocardiography, thallium stress testing and cardiac catheterization. Holter monitor data prior to, and after institution of ranolazine 1000 mg twice daily was compared. Patient tolerance and sense of well being after ranolazine was assessed.

**Results:**

Significant LVH was noted and obstructive coronary artery disease was ruled out by cardiac catheterization. Within two hours of the initial dose of ranolazine a marked decrease in ventricular ectopy was observed. Ventricular ectopy on Holter monitor decreased approximately 12 fold (23.8% of beats to1.9%) while supraventricular ectopy decreased approximately 7 fold (5.3% of beats to 0.8%). The decrease in ectopy was associated with an improved sense of well being.

**Conclusion:**

Ranolazine had rapid onset, potent anti-arrhythmic properties in the absence of obstructive coronary artery disease in a patient with LVH and may be an ideal agent in patients where few anti-arrhythmic options exist.

## Introduction

Malignant arrhythmias are a leading cause of morbidity and mortality in patients with, and without, ischemic heart disease [1-7]. In patients with left ventricular hypertrophy due to hypertension, ventricular arrhythmias are common and associated with an increased risk of sudden cardiac death in excess of the risk attributable to hypertension alone or presence of coronary artery disease [2-4]. The mechanisms of arrhythmias are varied and include enhanced automaticity, reentry and triggered activity from after depolarizations [5-10]. Triggered activity may be the most important mechanism in patients without cardiac ischemia [5-10].

Ranolazine is a novel anti-anginal agent, which inhibits the abnormal late inward Na^+^ current in ventricular cardiomyocytes [11]. This produces an energy-sparing effect and indirectly decreases diastolic intracellular cardiomyocyte Ca^++^ levels. This effect also improves membrane stability [11]. Ranolazine has also been shown to be a potent inhibitor of after depolarizations produced by a number of mechanisms [12-14]. As such, it could prove to have potent anti-arrhythmic properties in some arrhythmic situations, particularly those mediated by after-depolarizations. Additionally ranolazine inhibits IKr and prolongs repolarization without increasing the dispersion of repolarization [12-14].

In this case report, we describe the dramatic anti-arrhythmic effects of ranolazine in a patient with significant left ventricular hypertrophy and symptomatic ventricular ectopy and supraventricular ectopy.

## Case Report

A 72-year-old male presented to our office for evaluation of a one- month history of increasing exertional dyspnea, palpitations and fatigue. The patient had past history of hypertension, ulcerative colitis with mild associated anemia, prostate cancer, and mild sinus bradycardia. His medications included furosemide 20mg/day and amlodipine 5mg/day. His physical exam was unremarkable except for mild bradycardia (55 beats/min.), frequent ectopic beats and a soft systolic ejection murmur.

**Figure 1:**
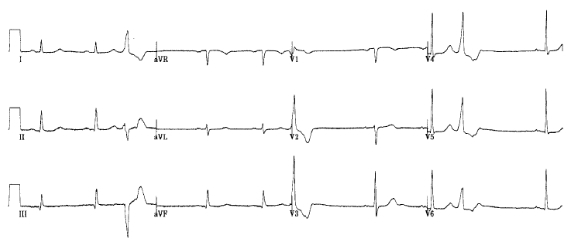
Electrocardiogram showing LVH and frequent ventricular ectopy

Initial workup included a normal chest x-ray, electrolytes, renal indices, and thyroid function studies. A hemogram confirmed his past history of mild anemia (hemoglobin 12.7 grams/dl) with a normal differential counts. The electrocardiogram revealed mild sinus bradycardia with frequent premature ventricular contractions (PVCs), non-specific ST-T wave changes and a corrected QT interval of 471 msec (Figure 1).

Transthoracic echocardiogram revealed concentric left ventricular hypertrophy (posterior wall thickness 15 mm, septal thickness 18 mm) without any out flow obstruction, left atrial enlargement, and grade II diastolic dysfunction. During the stress test, the patient was unable to tolerate exercise due to severe dyspnea and reached a heart rate of only 116 beats/min. Consequently the study was completed using an adenosine stress agent. Prior to terminating the exercise, moderate diffuse ST segment depression was observed in the inferior and anterior leads. His baseline electrocardiogram revealed frequent PVCs in a pattern of bigeminy. The PVCs decreased in frequency with exercise. The Thallium imaging was negative for ischemia and the ejection fraction was estimated at 66%.

24-hour Holter monitoring revealed frequent PVCs (Table 1). Ventricular ectopic beats made up for 23.8% of total beats. There were triplets and couplets present, but no runs of ventricular tachycardia. There was moderately frequent supraventricular ectopy as well accounting for 5.3% of total beats. There was a tendency towards bradycardia.

Because of his symptoms, frequent ectopy and concerns for "balanced ischemia" he underwent cardiac catheterization which revealed very mild non-obstructive coronary artery disease and a normal global left ventricular systolic function. The left ventricular end-diastolic pressure was elevated at 30 mm Hg. During the monitoring period prior to, and during the catheterization, the patient continued to demonstrate very frequent ventricular ectopy.

On the basis of the information obtained, the patient was felt to have hypertensive heart disease with the frequent ectopic beats impairing cardiac performance. We felt it was highly likely that triggered activity was the mechanism of his ectopy. Thus, shortly after the catheterization he was given a 2000 mg dose of ranolazine [15] as he recovered from the catheterization in a monitored setting. Within two hours of this dose, a marked reduction in ectopy frequency was observed and consequently he was discharged on a ranolazine dose of 1,000mg twice daily. A follow-up Holter monitor was arranged which revealed a dramatic reduction in ventricular and supraventricular ectopic activity (Table 1).  A slight reduction in average heart rate was observed without a decrease in minimum heart rate or increase in the longest R to R interval (Table 1). A repeat ECG showed absence of all ectopy and a corrected QT interval of 443 msec. 

The patient noted marked improvement of his presenting symptoms and consequently requested that we continue ranolazine, though the dose was later lowered to 1000 mg in the morning and 500 mg in the evening because of constipation.

## Discussion

In our patient with frequent symptomatic ectopy and significant left ventricular hypertrophy due to chronic hypertension, we observed a rapid and marked reduction in both ventricular and supraventricular ectopy with the anti-anginal agent ranolazine. Indeed within a couple of hours the ectopy went from being extremely frequent to very occasional. This occurred despite the absence of obstructive coronary artery disease indicating that this was a direct electrophysiologic effect of this medication. We have previously reported the ability of ranolazine to favorable effect ventricular ectopy in a case on non-ischemic cardiomyopathy in a rapid manner [16] as well idiopathic ventricular tachycardia in a patient without structural heart disease [17]. This case extends these observations to the setting of hypertensive heart disease complicated by significant left ventricular hypertrophy.

In patients with significant left ventricular hypertrophy, there are limited anti-arrhythmic options due to the risk of pro-arrhythmia [18]. Most anti-arrhythmic agents induce QT prolongation and increase the dispersion of repolarization, an effect worsened by the presence of left ventricular hypertrophy [19]. Ranolazine has minimal effects on the QT interval and does not increase dispersion of repolarization [13]. Ranolazine has no known pro-arrhythmic effects and consequently may be the ideal agent in such circumstances.

Although we feel that a reduction in triggered activity most likely explained the reduction in ectopy [12-14], we can not be certain as to the mechanism involved. In addition to decreasing triggered activity, therapeutic levels of ranolazine also inhibits IKr which slightly prolongs repolarization and decreases dispersion of repolarization [12-14]. These effects could make re-entry harder to become initiated or sustained.

Our patient also had relative bradycardia, likely reflecting sinus node dysfunction. This precluded us from initially trying a beta blocker for the ectopy.  Although we observed a slight decrease in the average heart rate with ranolazine between the two Holter monitors, no pauses or worsening of the minimum heart rate was observed.

## Summary

Ranolazine was remarkably effective in decreasing ventricular and supraventricular ectopy in a patient with significant hypertrophic heart disease secondary to chronic hypertension. More investigations into the anti-arrhythmic effects of this agent in man are warranted.

## Figures and Tables

**Table 1:**
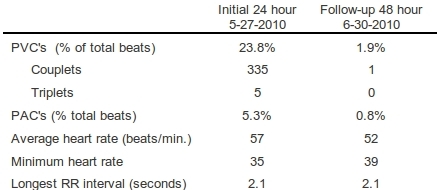
Effect of ranolazine on Holter data

PAC's: premature atrial contractions, PVC's: premature ventricular contractions
